# Impacts of Trace Metals Pollution of Water, Food Crops, and Ambient Air on Population Health in Zambia and the DR Congo

**DOI:** 10.1155/2022/4515115

**Published:** 2022-07-05

**Authors:** A. Muimba-Kankolongo, C. Banza Lubaba Nkulu, J. Mwitwa, F. M. Kampemba, M. Mulele Nabuyanda

**Affiliations:** ^1^Department of Plant and Environmental Science, School of Natural Resources, Copperbelt University, P. O BOX 21692, Jambo Drive, Riverside, Kitwe, Zambia; ^2^Unit of Toxicology and Environment, School of Public Health, Faculty of Medicine, University of Lubumbashi, Lubumbashi, DR, Congo; ^3^Faculty of Agricultural Sciences, Department of Zootechny, University of Lubumbashi, Lubumbashi, DR, Congo; ^4^School of Mines and Mineral Sciences, Department of Environmental Engineering, Copperbelt University, Kitwe, Zambia

## Abstract

Zambia and the DR Congo are situated in the central African Copperbelt, which is part of the Lufilian geological structure arc stretching over from Kolwezi in Katanga Province in the DRC to Luanshya in Copperbelt Province in Zambia. The area has large copper-cobalt deposits of which the extraction causes severe ecosystem damage due to pollution of water, food crops, and the ambient air negatively impacting population health. Contamination of drinking water for domestic use and foods (cereals, roots and tubers, vegetables, and fruits) was determined by assessing the contents of trace metals including Mn, Ni, Pb, Zn, Co, As, U, Cd, and Cu and through a questionnaire for environmental damage. Food samples were analyzed by inductively coupled argon plasma/optical emission spectroscopy (ICP-OES), while water and urine samples were analyzed by inductively coupled argon plasma mass spectrometry (ICP-MS). Concentrations of Ni, Pb, and Cd were higher in almost all food crops, although Cu was more in samples of *Cucurbita maxima* and *Amaranthus hybridus*. Mean contents (*μ*g/L) of Mn, Zn, Cd, Pb, and U were, respectively, 5,454.6, 2552.2, 138.7, 39.7, 2361.1, and 21.4 in the DRC and 108.9, 543.3, 0.3, 0.2, 1.5, and 0.5 in Zambia, being significantly higher and always far above World Health Organization maximum limits in the DRC. Urine samples taken only from the DRC contained trace metals with children's samples being more contaminated than adult ones. Our results conclusively echo the most critical challenges of toxic pollutants causing numerous health issues among the population. Given an outcry among households adjacent to mines about land degradation and food spoilage, and health problems over years, joint efforts are needed from public and private sectors for stringent mining exploitation monitoring for sustainable governance to protect the environment and ensure food and nutrition safety, and population well-being in Zambia and the DR Congo.

## 1. Introduction

Zambia and the DR Congo (DRC) are situated in the central African Copperbelt region, which is part of the Lufilian geological structure arc that stretches over more than 500 km from Kolwezi in the former Katanga Province in the DRC to Luanshya in Copperbelt Province in Zambia [1]. The arc contains large reserves of copper and cobalt minerals [2], which represent the mainstay of the two countries' economy [3–8].

With about 28% contribution to the GNP and 70% export value [6], mining exploitation remains the main source of DRC's foreign currencies [6, 7]. Similarly, the mining sector is Zambia's most sensitive economic sector owing to its linkage to the export market, and copper contributes about 70% of foreign earnings [9]. The mining industry only contributes about 9.90% to GDP, 77% to export earnings, and 27.77% to country employment [10]. Besides public and private industrial mining, artisanal mine productions are also widespread [11–15] and often work in the form of cooperatives of artisan miners—also known as formal artisanal mining—leading to the establishment of a middle class of Congolese and Zambians contributing to the integrated development of grassroot communities. They operate on industrial mining concessions using nonstandard extraction practices such as sieving the soil in rivers, thereby causing severe environmental pollution. There are currently numerous public, parastatal, and private companies and informal artisanal mining operating in the mining sector in Zambia and in the DRC [7, 14, 15]. Brummett et al. [16] recorded the reduction in water quality through mining pollution as a major threat to freshwater ecosystems, and Banks et al. [17] and Pulles et al. [18] noted that discharges of contaminants in water resources constitute the main environmental challenges to most mining exploitations.

Much of the sub-Saharan Africa including the central Africa Copperbelt comprises more poor rural communities that depend on agriculture for livelihoods and as an important source of commodities' exports and intraregional trade [19]. The agricultural sector in the region is dominated by crop production mainly by smallholder farmers—because of the climate change that has exacerbated weather variability—over-depending on rainfed agricultural production of roots and tubers, plantains, maize, rice, groundnuts, beans, sorghum, millets, and vegetables [19–22]. Estimates from the World Bank [23] indicate that the value addition to GDP attributable to agriculture in the DRC and Zambia is 42.9% and 21.6%, respectively. About 75% of the population is engaged in agriculture, largely subsistence farming that contributes about 60% to the overall production output [24]. Nevertheless, the performance of agriculture in the central Africa Copperbelt region in terms of food production and security has been lacking far behind mainly due to recurrent prolonged droughts, making food insecurity a major challenge [25, 26]. Moreover, food security is compromised by the degradation of farmlands and the environment resulting from mineral extractions [27], often culminating in food shortages and famine. The community's inhabitants are perpetually prone to hunger, malnutrition, and poverty. Contaminants from mines and metallurgic industries pollute water, soil, and the ambient air affecting the growth pattern and well-being, especially for children. For decades, mining activities including construction, exploitation, and maintenance have caused severe damage to the regional ecosystem resulting in land-use change and associated negative impacts on environments such as deforestation, erosion, alteration of agricultural soil properties, contamination of local streams and wetlands, and an increase in noise level, dust, and smoke plume emissions [27, 28]. Pollution of the ambient air from industrial activities caused mainly by pollutants being transported over long distances after their release into the environment has also been the source of health concerns. Other negative impact communities living on fringe of the mines endure include the impacts on rainfall pattern modification becoming less and often starting late in the year leading to the decline of the forest cover and considerably decreasing forest resources used for their well-being [27, 29, 30].

Health threats posed to residents from prolonged exposure to toxic trace metals and metalloid can only be determined by their daily dietary intake through ingestion and inhalation pathways. Individuals' life span trend and their well-being could also serve as broad indicators for their physiological food uptake, digestion, and storage as well [31, 32]. Numerous studies have been carried out in the DRC in relation to human body accumulation of pollutants and showed high amounts of pollutants in the blood and urine of inhabitants in the mining areas including mineworkers, residents of an urban neighbourhood that had been transformed into an artisanal cobalt mine, and children with strong evidence of exposure-related oxidative DNA damage [15, 33, 34]. In contrast in Zambia, only investigations in children suffering from lead poisoning are well established in the plomb mining area in Kabwe town that has left a legacy of significant environmental pollution [28, 35]. Aside from the threat of increased and prolonged exposure to trace metal contaminants in food crops that were sampled in this study, we also acknowledged that local communities in the two countries have a varied and diversified food staples depending on ethnicity, culture, and where they live. Hence, our study could have also included foods such as fresh, frozen, and smoked fish, fresh and smoked meat (goat, chicken, beef, pork), eggs, eggplants, rape, okra, poultry, caterpillars, grasshoppers and flying termites, and lentils that are commonly utilized in the diet to fully evaluate the body burden of trace metals among the devastated populations. Although ambient air pollution has been thoroughly investigated by others [15, 36–40], data on the identification and distribution of metal particle size are critically needed both from emission sources and in the ambient air. Particle size data on trace metals can be used to appraise the efficiency of emission control devices, evaluate the inhalation hazard, determine the timing of metals in the atmosphere, and assess visibility reduction and for a wide range of other applications including understanding atmospheric transformations.

Given the enormous scale of damage to the ecosystems resulting from mineral extraction and operations of metallurgic industries that generate hazardous wastes containing dissolved toxic metals over the years, there has been an outcry among households adjacent to mines about land degradation, water and air pollution, and food spoilage. This study was carried out to investigate the perceptions of inhabitants living around mining areas and other stakeholders as regards the environmental and social impacts on the communities from industrial activities with the objectives to improve the population health and livelihood.

## 2. Materials and Methods

### 2.1. Study Sites

**Figure 1:**
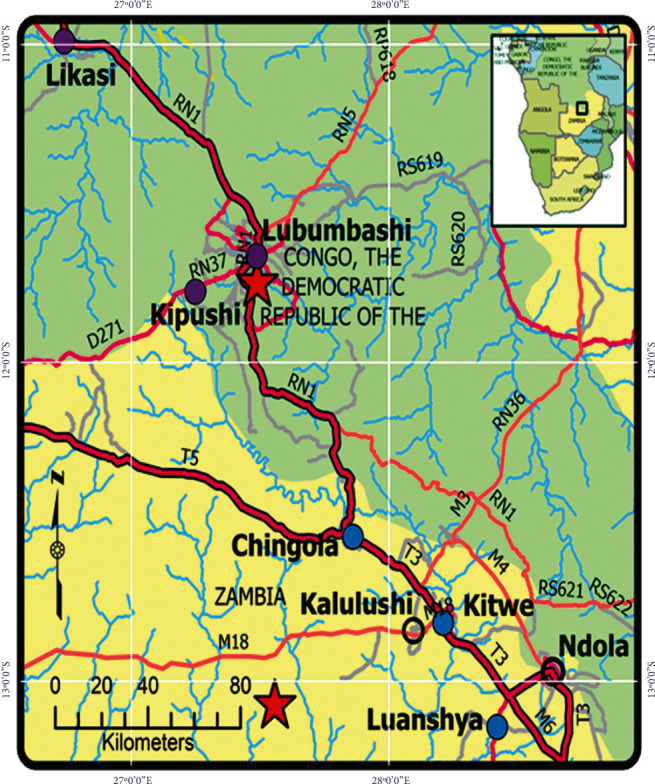
A geographic map of different locations with mineral mining cities and districts surveyed in the region with Kipushi Territory, Likasi District, and Lubumbashi City in the DR Congo 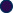, and Chingola, Kitwe, and Luanshya Districts in Zambia 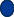 for samples collection and interview with community members.

This study was concentrated in the copper-cobalt producing areas of Zambia and the DRC (Figure 1) from September 2010 to September 2012 to enable a cross-country assessment of landscape pollution and impacts on population health. They were Haut-Katanga Province (Lat. 11°39′52.84″S, Long. 27°28′46.38 E, Alt. 1239 m) in the southeastern DRC where Gécamines—the state mining company—with its subsidiary industries have huge concessions covering approximately 30,000 km^2^ around the towns of Likasi (Central Group), Kipushi (Southwest Group), and Lubumbashi (East Group), and the Copperbelt Province in Zambia (Lat. 13°03′25.23″S, Long. 27°32′58.50″E, Alt. 1247 m) where various companies including Konkola Copper Mines (KCM), Nchanga Mines, Mopani Copper Mines, Chambishi Metals, Roan Antelope Mining Corporation, and Luanshya and Kansanshi Mines extract minerals. KCM, one of the largest mining companies belonging to Vedanta, operates underground mines and open-pit mines and metallurgical plants with operations located on one of the highest-grade copper seams at Nchanga, Konkola, and Nkana in Kitwe and Nampundwe.

A total of three sampling locations were specifically selected within each country based on recent proliferation of mines and previous reports on environmental contamination from plant effluents, spills, and tailings dam discharges and to have a wide distribution of sampling sites across the two countries. They were cities of Lubumbashi and Likasi, and Kipushi Territory in Haut-Katanga Province in the DRC, and Chingola, Kitwe, and Luanshya Districts in Zambia. Within each location, several sites were identified for sampling either in the proximity of intense mining and metallurgic activities or far away as controls based on the premises of past farmland degradation, potential degrees of high or low exposure, easy accessibility, and avoiding health risk due to possible irradiation from dangerous mining areas. They included Kabecha, Tshansansa, and Kasongo sites in Lubumbashi; SNCC-Shituru, Gécamines-Shituru, and Panda cities, and Kansalabwe village in Likasi; and Mungoshi and Douane areas, Betty City, Kampemba farm, and Nkumanwa village in Kipushi for the DRC. In Zambia, they were Shimulala, Heleni-Kasenji, Kalipenta, Pule, and Chama villages in Chingola; Roan area, and Mpatamatu and Mwansa villages in Luanshya; and Chankalamu village and Wusakile and Nkana West compounds in Kitwe.

### 2.2. Approaches to Sample Collection and Analysis

#### 2.2.1. Collection and Analysis of Food Crop Samples

Prioritised samples that were at the full stage of development were collected with permission in residents' gardens following the sites mapping around mine areas and far away and local harvesting practices, considering grains, leaves, stems, tubers/roots, and bulbs as plant parts for laboratory analysis. Precautions were taken to have both women and men during the briefing, which focused on hazardous chemicals for which long-term exposure might have potential effects on their health. After harvest, samples were immediately kept in paper bags, labeled accordingly, and kept in a refrigerator. In total, the samples included root and tuber crops (*n* = 48), leafy vegetables (*n* = 143), fruits (*n* = 51), bulbs (*n* = 5), and other plants (*n* = 33) that are also locally consumed. Out of these, 205 were from areas of intense mining and metallurgic industry activities, whereas 75 were derived from areas distant from these activities. Collected samples were pretreated individually by rinsing twice in running tap water and 3-4 times with sterile distilled water to remove any soil particles and other plant debris from fields and blotted dry before slicing into small pieces using a sterile knife. They were then oven-dried at 70°C to constant weight for 48 hours and separately homogenized by mechanical grinding using sterile porcelain mortars and pestles made from China. The weight of each sample powder was recorded before wrapping it in minigrip bags, which were maintained at room ambient temperature for further use. Thereafter, study samples were air-dispatched to soil and occupational and medicinal laboratories in Belgium for analysis.

As described by Muimba-Kankolongo et al. [31], ground oven-dried powder samples of various food crops were crushed and sequentially digested in HNO_3_ (3 successive runs of dissolution in HNO_3_ (1 mL Suprapur 70%) followed by evaporation to dryness for 4 h at 110°C) and by microwave heating (250 W × 5 min; 400 W × 5 min; 650 W × 10 min; 250 W × 5 min) and then left to cool, and contents were filtered through Whatman filter paper no. 42 and kept in a final solution of 2% HCl and analyzed for total metal content by inductively coupled plasma/optical emission spectroscopy (ICP-OES, Perkin-Elmer Optima 3300 DV, Norwalk, CT, USA) in the Louvain Centre for Toxicology and Applied Pharmacology (Université catholique de Louvain, Belgium). At concentrations of b5 *μ*g·Co/L in the digest, measurements were made by ICP-MS (Thermo X Series I) in He mode using Ga internal standard. Certified reference material NIST SRM-1573a (tomato leaves, National Institute of Standards and Technology, Gaithersburg, Germany, certified value = 0.57 mg Co/kg; measured value = 0.54 ± 0.01 mg·Co/kg) was included in duplicate with each analysis. Contents of 20 different trace elements and metalloids were determined, among which those of major concern in foods [32–34] included Mn, Ni, Pb, Zn, Co, As, U, Cd, and Cu. Metals and metalloids released by the digestion were added to NH_4_I solution, extracted by MIBK-TBP (80 : 20), and then stripped with H_2_O_2_ (5%), HNO_3_ (0.5%), and NH_4_H_2_PO_4_ (0.1%). Quantification was done by injecting aliquots of sample solutions (10 *μ*L) into a carbon rod atomizer of an atomic absorption spectrophotometer with a variable detector set at 0.5 absorbance unit full scale and the results expressed in mg/kg of dry matter of each crop species.

#### 2.2.2. Collection and Analysis of Water Samples

**Figure 2:**
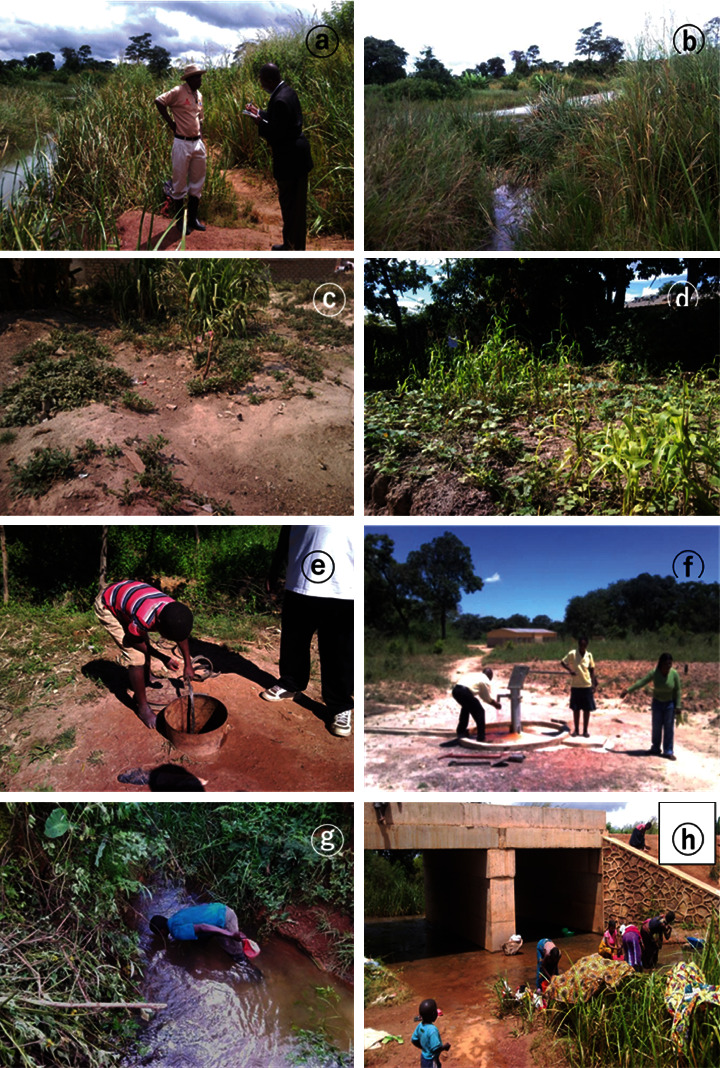
Stunted crops in gardens and surface and underground water of poor quality due to mine and metallurgical industries waste: interaction with the affected population showing (a) discussion with a concerned member of the community; (b) vegetation dying around a polluted river bank close to a mine site in Lubumbashi in the DRC; (c) stunted maize intercropped with pumpkin vegetables in Luanshya in Zambia; (d) sweetpotato in poor growth in Lubumbashi in the DRC; (e) a young boy fetching contaminated underground water from an open borehole for use at home in Lubumbashi; (f) a closed borehole with a pump for many village residents in Kitwe in Zambia; (g) a woman relying on surface polluted drinking water from a nearby gallery forestry source; and (h) women washing clothes and bathing in a polluted river in Lubumbashi in the DRC.

For water samples, every effort was made to maintain a high degree of cleanliness for all equipment including bottles, containers, and rope and filtering equipment. At each location, water samples were collected in areas near mining activities or metallurgical factories (*n* = 43), but also in areas far from mining or mineral processing sites (*n* = 23) from rivers (*n* = 13), domestic wells or boreholes (*n* = 43) and taps (*n* = 23) in the districts of Chingola (*n* = 21), Kitwe (*n* = 11), and Luanshya (*n* = 10) in Zambia and in the cities of Lubumbashi (*n* = 6) and Likasi (*n* = 19), and the Kipushi Territory (*n* = 9) in the DRC always between 8 and 11 h. There were 205 samples collected in the fringe areas of mining activities and 75 control samples from areas far from mining and metallurgical industries. For surface rivers, the sampling was always at midstream or in the main flow and away from slumping and scouring effects found near the banks. The sampling bottle was uncapped immediately before sampling and plunged into the river with the opening facing upstream into the current until it was filled. Then, it was lifted out of water, decanted for a small amount if required, and recapped immediately ensuring hands did not come into contact with the insides of the bottles or caps. Adequate water quantities were obtained from a full flow out of faucets/taps after taps or pumps were left to run for at least two minutes to clear the line (Figure 2). Water was slowly filled in 40 mL clean polystyrene bottles with leak-proof screw caps (Plastiques Gosselin, Hazebrouck, France) to below the container rim before tightening the lids securely. In the deep dug boreholes or wells, water was obtained using tins cut on one side or plastic containers connected to long wooden canes with wire or rope (Figure 2) and filled in the polystyrene bottles. Samples were then pipetted into 1.5 mL Eppendorf tubes using micropipettes and transported in cooler boxes to the laboratory to be stored in the refrigerator at −20°C to retard potential chemical and biological changes before shipping in sealed cooler boxes with ice packs for analysis. Collected samples were filtered (0.45 *μ*m) and measured by ICP-OES. The NIST certified water was included (certified value = 27 *μ*g Co/L, measured value = 26 ± 1 *μ*g·Co/L).

Prior to analysis, water samples were filtered using a *μ*m micropore membrane filter (0.45 *μ*m) to avoid clogging the atomizer burner and then trace metal contents were quantified following the description by Muimba-Kankolongo et al. [31]. In particular, they were analyzed using inductively coupled argon plasma mass spectrometry (ICP-MS) with the lowest detection limit of 0.5 ppb (Perkin-Elmer Optima 3300 DV, Norwalk, CT, USA). The NIST-certified water was included (certified value = 27 *μ*g Co/L, measured value = 26 ± 1 *μ*g Co/L). Detection limits for all samples are calculated as twice the standard deviation of 6 preparation blank sample measurements as described by Cheyns et al. [39]. For values below the detection limits, half of the detection limit of each trace element was considered and the results were expressed in *μ*g/L of water.

#### 2.2.3. Collection and Analysis of Urine Samples

Urine samples were obtained only from the DRC and not in Zambia because of some administrative issues with the Ministry of Health. Donors of urine samples included females and males among them there were children and adults. The youngest was 2 years old, while the oldest was 58 years old. The collection was from community members composed of infants and children over 2 years old (*n* = 9), adolescents (*n* = 4), adults (*n* = 13), and the elderly (*n* = 3) in Kabesha and Kawama locations in Lubumbashi following procedures previously described by Banza Lubaba et al. [37]. The collection was during the whole day often with assistance from local health personnel, and participants—both parents and children—were duly informed of the need for urine in the study before samples were collected and their participation was purely based on their willingness. During collection, instructions were provided to participants on how to perform a proper collection to avoid external contamination of the urine. After collection, an aliquot of 5 N hydrochloric acid was added to prevent decomposition and loss of metals by precipitation and then immediately stored in a cooler box and taken to the laboratory for storage in a refrigerator until transportation to Belgium for analysis, using commercial flights. Only containers from subjects with sufficient urine for analysis were considered. Although the focus was on urine collection, additional information about the characteristics of each participant such as anthropogenic indices (birth date, age, sex, weight), demographic indicators (time spent at site, past and present occupation), medical history (particular symptoms, general health status), vital signs (heart rate, pulse), and smoking and alcohol intake patterns were recorded to track possible levels of intoxication.

Solubilized urine samples were analyzed in the Louvain Centre for Toxicology and Applied Pharmacology (Université Catholique de Louvain, Belgium) using blind analysis as previously published [37, 41]. Twenty-four trace metals were quantified, in all samples, by means of inductively coupled argon plasma mass spectrometry (ICP-MS) on an Agilent 7500ce Instrument. Briefly, urine specimens (500 *μ*L) were diluted quantitatively (1 + 9) with a HNO_3_ 1% and HCl 0.5% solution containing Sc, Ge, Rh, and Ir as internal standards. Briefly, urine specimens (500 *μ*L) were diluted quantitatively (1 + 9) with a 1-butanol (2% w/v), EDTA (0.05% w/v), Triton X-100 (0.05% w/v), NH_4_OH (1% w/v) solution containing Sc, Ge, Rh, and Ir as internal standards. Hg and Pd were analyzed using no-gas mode. Limit of detection (LoD) and limit of quantification (LoQ) were calculated as three and nine times, respectively, the standard deviation of the blank signal. Using these ICP-MS validated methods, the laboratory has obtained successful results in external quality assessment schemes organized by the Institute for Occupational, Environmental and Social Medicine of the University of Erlangen, Germany (G-EQUAS program), and by the Institut National de Santé Publique, Québec (PCI and QMEQAS programs). As an illustration, all results, except Li, In, and Pd not included in the QMEQAS program, were associated with a z'-score comprised between −1.00 and + 1.00 for the round QMEQAS 2012–01 processed at the time of the study sample measurements. Furthermore, it should be mentioned that the laboratory possesses an ISO15189 certification for the measurement of 20 metals in urine (Be, Al, V, Cr, Mn, Co, Ni, Cu, Zn, As, Se, Mo, Cd, Sn, Sb, Te, Ba, Tl, Pb, and Bi). Urinary creatinine was also determined using a Beckman Synchron LX 20 analyzer to correct for urine dilution (Beckman Coulter GmbH, Krefeld, Germany) to establish whether or not participants' kidney was normally functioning. Final metal concentrations were then corrected for creatinine by dividing the concentration of urinary trace elements of each heavy metal and metalloid by the creatinine concentration, and the results are shown as *μ*g/g creatinine. Creatinine is a chemical waste product produced through one body's metabolism that indicates whether the kidneys are functioning normally by filtering creatinine and other waste products out of the blood in the body through urination. Thus, urine creatinine will measure the concentration of chemicals of interest in urine [42, 43].

#### 2.2.4. Population Knowledge of Pollution Impacts

The study investigates here environmental and social impacts and the residents' perception of the impacts of mining activities on their communities in the light of the numerous promises and prospects that mining is said to provide for communities. Respondents for participation in the study were done with the objectives to determine social, economic, and health issues communities are facing in relation to environmental pollution from mining and metallurgic industries. Diverse actors were selected for an interview at the village, district, provincial, and national levels. At the provincial and national levels, they included local government officers namely personnel in ministries of health, environment, health and forestry, and representatives of the mines. Respondents at the district level included leaders of political parties; mine workers' affiliations; residents around mining; institutions responsible for the environment; and NGOs and CBOs. For the local communities, sample households from villages were randomly selected at regular intervals of about 10 km up to 50 km from the fringes of the town and in areas of industrial mining operations. A participatory rural appraisal approach was adopted focusing on both men and women as beneficiaries of numerous resources and traders and artisanal miners, although they might have different perceptions of mining benefits and negative impacts.

Adults in targeted communities were subjected to semistructured interview techniques integrating the probing open-ended questionnaire and a face-to-face discussion. The questionnaire was structured into thematic components based on relevant actors to yield the most reliable information about mining impacts on the surrounding population and natural resources. Questions were asked to capture the knowledge levels of mines' negative impacts on the community's farmlands, food and water quality, and potential illnesses inhabitant face. The questionnaire was also designed to get possible suggestions they could provide on mining management and governance strategies for environmental protection. Face-to-face interviews with stakeholders were done either at households, workplaces, or in the field of growers to yield the most reliable information without the participation of mining and industrial companies. They dealt with the availability of alternative water sources, the importance of water for livelihood, consumption of homestead garden-grown food, food and livelihood security, time spent in the area, and impacts of mines on well-being and mines' corporate social responsibilities to the community. Questions also included any alternative to the water obtained near homesteads, type of normal life if the water source was contaminated or cut off, time of consuming food grown around homesteads or village, factors contributing to food security and secured livelihood, impacts of mining activities on livelihood, and the role that mining companies can play in better community livelihood. Care was taken to provide opportunities to respondents to indicate concern about environmental pollution voluntarily. The response rate to the interview was 100%—reflecting the high interest of inhabitants who participated massively—and the data collected were representative of the entire community population.

## 3. Data Analysis

Study data were analyzed using a Statistical Package for Social Sciences (SPSS version 19, Chicago, IL, USA), and Fisher's test (*p* ≤ 0.05) for a one-way analysis of variance (ANOVA) was used to compare variances between trace metal concentrations across countries, districts, and sites in districts within each country and their interactions. Some data were logarithmically transformed for statistical analysis after testing the original data for normality.

## 4. Results

### 4.1. Contamination of Food Crops

The results of toxicological analysis of plant tissues from crops utilized as food show high levels of contamination by heavy metals and metalloids. Concentrations of pollutants were significantly different (*p* < 0.001) between the DRC and Zambia and between the six locations surveyed in the two countries (31 in Table 2 and Supplementary Data Table D). The distributions of the concentrations of the nine trace elements in food crops from Zambia (*n* = 128) and the DRC (*n* = 145) ranged several magnitudes between the lowest and highest concentrations (Table 2). Concentrations were significantly higher in plant samples obtained from the DRC than in those obtained from Zambia, for all elements except Mn. Contents of As were found to be below the limit of quantification (LOQ) but higher in crop samples from the DRC than from Zambia.

The Kruskal–Wallis test followed by Dunn's multiple comparisons test of trace element geometric means and their ratios is shown in Supplementary Data D. Statistically significant differences were found for six elements (Co, Cu, As, Cd, Pb, and U) between concentrations of trace metals in crops growing near mining compared with concentrations in sites remote from mining, with their geometric means ranging from about 2 for As to more than 5 for Co.

Except for arsenic (As), whose content was significantly higher in Kipushi Territory in the DRC, overall, the crops grown in Lubumbashi were exceedingly contaminated than those from Likasi and Kipushi and those from any district in Zambia. Generally, the concentrations of nickel (Ni), lead (Pb), and cadmium (Cd) were higher in almost all food crops that were analyzed, although copper (Cu) was more in samples of *Cucurbita maxima* and *Amaranthus hybridus*. However, although Cd and lead are all present in both countries, their contents greatly exceed the allowable standard values for food plants in the DRC.

### 4.2. Water Contamination with Trace Elements

Statistical analysis of the data clearly indicates considerable variation in the levels of trace metals between countries and the various sites where the samples were derived. ANOVA revealed significant differences (*p* ≤ 0.001) in the concentrations of all trace metals and metalloid recovered in water samples from the DRC and Zambia. Highly contamination was recorded in the DRC (31 in Table 1 and Supplementary Data Table B). The distributions of the concentrations of the nine trace elements in water from Zambia (*n* = 42) and the DRC (*n* = 35) ranged several magnitudes between the lowest and highest concentrations (Table 1). Concentrations were significantly higher in water samples obtained from the DRC than in water from Zambia, for all elements except Zn. As a result, ratios of median value concentrations obtained in the DRC were always above WHO guidelines for drinking water. The strength and direction of association between contents of trace metals in water samples using Spearman's correlation analyses (Supplementary Data Table B) showed a weak correlation between concentrations of trace metals from Zambia and a strong one in samples from the DRC.

Mean contents (*μ*g/L) of trace metals such as Mn, Zn, Cd, Pb, and U were, respectively, 5,454.6, 2552.2, 138.7, 39.7, 2361.1, and 21.4 in the DRC and 108.9, 543.3, 0.3, 0.2, 1.5, and 0.5 in Zambia. Concentrations of trace elements in water from boreholes were significantly higher in Likasi city (average = 20,791.4 *μ*g/L) than concentrations in any other location surveyed in the DRC. In contrast, in Zambia, concentrations of trace elements were only high in tap water from Chingola District (average = 223.5 *μ*g/L) than the concentrations in water from either Kitwe District or Luanshya District, but all concentrations in water were still far below the concentrations of metals in water from the DRC.

### 4.3. Trace Elements of Heavy Metals and Metalloid in Urine

**Figure 3:**
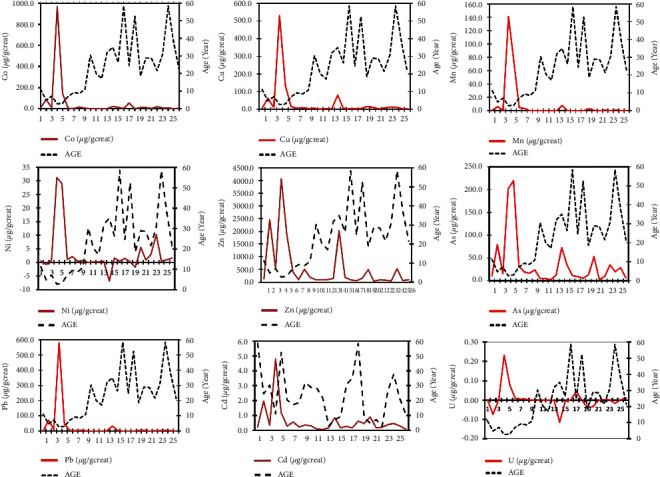
Elements of trace metals and metalloids (*μ*g/g creatinine) in urine samples from inhabitants in Kabesha and Kawama villages in Lubumbashi city, the DR Congo with almost all metals (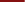) being greater in younger than elderly people (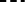).

Laboratory analysis of samples revealed abnormally high contents of trace elements of heavy metals and metalloid in all participants' urine (Figure 3) with the concentration ranges (*μ*g/g of creatinine) for Mn (0.03–140.94), Co (0.5–969.13), Ni (−0.01–31.17), Cu (2.63–531.35), Zn (40.36–4079.08), As (2.70–219.95), Cd (0.07–4.79), lead or Pb (0.64–580.87), and U (−0.002–0.24). Overall, the levels of trace metals in the urine from children were higher than those in urine from adults, with exceedingly higher concentrations of all trace metals (e.g., 140.9 for Mn, 969.1 for Co, 31.2 for Ni, 531.3 for Cu, 4,079.1 for Zn, 201.9 for As, 1,761.5 for Mo, 4.8 for Cd, 580.9 for lead (Pb), and 0.2 for U·*μ*g/g creatinine) being recovered in the urine from one subject (a 2-year-old boy).

### 4.4. Population Reaction to Safety Issues

Although, inhabitants of the central Africa Copperbelt region acknowledged positive economic and social benefits from mines and metallurgic industries for their communities (Table 1)—such as sources of employment and roads, schools, and medical facility construction and maintenance—as part of their corporate social responsibility. However, these have been overshadowed by numerous negative effects on their livelihood. Our observations in various communities reveal that surrounding farmlands for agricultural production were degraded through soil erosion, loss of litter layer, acidification, and siltation, and both surface and underground water were of poor quality as a result of deposition and leakage of toxic dust and granules causing physical disturbance.

Furthermore, mining extraction and industrial operations were perceived to have negatively impact on the air quality, the stocking of valuable plant species including vegetation cover, soil, and the environment (Table 2). Industrial activities have considerably depleted the natural resources decreasing several forest products that are vital to their welfare. Of the surveyed residents, 100% stated that forest products including foods—wild fruits, vegetables, mushrooms, edible insects, honey, roots and tubers, and bulbs—and medicines, which sustain their livelihood, have become scarce. Moreover, the noise pollution from increased traffic of heavy mining trucks transporting copper and cobalt ores and other mine equipment, as well as mining blasts, was also indicated as matters of public concern—in particular for the residents close to mining sites in both the DRC and Zambia, imposing intolerable stress upon the community. Numerous other negative impacts of industrial exploitations were also highlighted during the interview including illegal settlement of fired miners due to a weak local economy, marginalization of community members for employment, and violation of human rights, especially for women.

Although artisanal miners are aware of their exposure to toxic metals such as uranium, they simply ignore the nature of any disease harm they might develop from prolonged exposures. They work without protective equipment by digging and transporting the ore either on bicycles or on the head for cleaning in nearby rivers. Their main complaints concerned the hard labor they perform, joint pain, chronic cough and colds, and more importantly the low income they receive from low price of their products. Inhabitants stated further that they have observed adverse health effects including eye and nose irritation, blood lead and cadmium intoxication, diarrhea, pneumonia, HIV/AIDS, cancer, tuberculosis, coughing, asthma, and sore throat and others such as skin tingling and persistent body fatigue.

When smoke plume from mining or metallurgic factories is emitted in the sky, people must hold their nose and mouth to avoid breathing contaminated air. They claimed that the dust from tailings dams and unpaved roads, and particularly emissions from smelter plants considerably, affects the air quality and constitutes the main elements exposing them to health hazards. Children's urine turns blackish at certain times and there are even cases of congenital malformations.

## 5. Discussion

Trace elements of selected heavy metals, namely Mn, Ni, Pb, Zn, Co, U, Cd, and Cu and one metalloid (As), that are often discharged from mine extractions and operations of metallurgic industries were recovered at various concentrations from water and numerous food crops commonly used for livelihood by the population in Zambia and the DRC. Their contents were exceedingly higher in the DRC than in Zambia and varied considerably depending on the sites assessed.

The contaminated agricultural food crops often grow and yield poorly with low-quality harvestable products because of soil acidification and siltation caused by activities of mining and metallurgic industries. Mean values of trace metals recorded in consumed food crops—expressed in *μ*g/g of dry matter—were significantly greater in the DRC than in Zambia, pushing inhabitants to believe that governments in the two countries are accountable for the situation as they are more interested in money than environmental health [28, 44].

It is generally well established that pathways of people's exposure to metals involve their ingestion (drinking or eating) or inhalation (breathing) [45]. Average values of trace metal concentrations recorded in water samples—expressed in *μ*g/L—were 5454.6 for Mn; 9530.4 for Co; 756.7 for Ni; 26113.8 for Cu; 2552.2 for Zn; 138.7 for As; 39.7 for Cd; 2361.1 for Pb and 21.4 for U and 108.9; 9.3; 2.4; 174.7; 543.6; 0.3; 0.2; 1.5; and 0.5, respectively, for the DRC and Zambia, raising vast and alarming concerns, especially for a high plumb (Pb) concentration in tap water from the DRC considerably well above the standard guideline value of 10 *μ*g/L. These results conclusively revealed that trace elements have considerably polluted water and vegetal species throughout the central Africa Copperbelt region. Their concentrations are higher, particularly in the DRC, than the recommended values in drinking water and food commodities [32]. Our results are consistent with other previous findings [37–39, 46–48] that environmental pollution from mining and industrial industries in the DRC and Zambia commonly occurs and destroys the environment and the natural resource base that are crucial to the people for their daily survival either for farming or for edible foods from forests [27].

Due to climate change in particular, groundwater resources often operated in urban areas by public networks are no longer able to cover, for a long period, the needs of the galloping population [49]. The use of surface water (springs, rivers, lakes) and underground water (open and closed boreholes) seems to be an alternative solution to provide the necessary minimum drinking water and potable water. The direct or indirect discharge of wastewater resulting from mining and industrial activities, without purification, in various areas often pollutes water tables, which are important for domestic purposes. The toxicity of heavy metals and metalloids contaminating water and agricultural products to which people are generally exposed is well known [50]. However, in many countries in Africa including the DRC and Zambia, there are not adequate regulatory provisions that stipulate threshold values or upper limits of trace elements of metals in food crops and water intended for human consumption [51]. Exposure and health issues from most environmental hazards are unevenly distributed within each country and often affect the most vulnerable people [52]. Some toxic trace elements recovered from water and food crops in this study, including arsenic, cadmium, copper, lead, and nickel, are known to enhance the risk of cancer in humans [50, 53] and can cause severe ill health after prolonged periods of exposure. Acceptable maximum limits of trace metals in water for domestic use have been set [53–55]. On basis of these limits, the concentration values for all trace elements detected in water samples from the DRC are considerably higher than the permissible recommended limits.

During the study period, it was evident that industrial effluents from waste dumps were released all over the landscape in addition to the spillage of mine waste through broken pipes, causing water flooding and erosion. Similarly, smoke plumb containing toxic chemicals was discharged into the atmosphere from the mining and metallurgic industries destroying the surrounding vegetation and polluting both surface and underground water [29, 38, 40, 56]. Although the enforcement and monitoring of obligatory compliance regulations of mining and metallurgic industries lie on hands of state governmental agencies such as the Zambia Environmental Management Agency (ZEMA) and the Labor Administration and the Labor Inspectorate of the DRC's ministry of labor that regulate and enforce laws, the responsibility on environmental protection in the concessions of mining and metallurgic industries remains with the owners to fully and correctly implement the submitted environmental management and monitoring plans.

Although owners of mines and metallurgic industries carry out some corporate social responsibility for the communities, it was, however, felt during the study that this was not enough. Almost all respondents across the surveyed villages have recognized that mining operations have negatively impacted their households' livelihoods. The vegetation was dying, and surface and underground water (e.g., rivers, wells, and taps) were heavily contaminated through suspended sediment load causing water to be of high levels of turbidity due to its cloudiness. Because the sediment deposition from industrial effluents includes particles of toxic chemicals, water quality is compromised mostly becoming unsafe for human uses. They recognized that the health of most people, including children and adults, especially those from low-income families, paints a rather unpleasant picture. They frequently display acute symptoms of malnutrition including stunting and being underweight. Most of them suffer from numerous sicknesses such as cancer, respiratory difficulties, and others, accounts that were also confirmed by local health officials and which agree with previous studies [27, 46]. Moreover, inhabitants highlighted the health effects on their auditory organs, blood pressure (cardiovascular system), and physiological well-being due to noise pollution from mining operations.

The laboratory analysis of some inhabitants' urine conclusively shows that all urine samples are contaminated with trace elements of heavy metals and metalloid whose concentrations were adjusted using levels of renal creatinine (*μ*g/g creatinine). Creatinine was determined to establish the contents of chemicals through renal evacuation [42, 43], which would be an essential predictor of accumulating or releasing toxic compounds from the body. Concentrations of trace elements were found more in children's urine than in adults, findings that are comparable to other previous studies in the area [37, 39, 57]. Children constitute the most vulnerable populations impacted by toxic metals than adults [53, 57]. Children are especially at exposure risk due to their continual increased hand-to-mouth activity, perpetual playing on the ground containing trace metals, and higher relative gastrointestinal absorption of elements, which they retain more readily [57]. However, we acknowledge that urine samples were obtained only from one country and from a small number of subjects not representative of the population in the area covered in the study. We are also certain from other previous studies [37, 39] that the contents of heavy metals and metalloid in renal functioning of a wider potential community sample will not depart from the findings in the current study.

Overall, the findings showed that concentrations of trace metals recorded in the DRC—except for Zn and Mn whose values were also high in food crops in Zambia—were largely above those in Zambia, while the two countries lie in the hinterland of copper-cobalt mining and share similar ore resources. The obvious discrepancy is for Zambia, constant environmental monitoring is performed to ensure conformity to established regulations and that the public officers involved have not been compromised by personal conflicts of interest in the mining sector [58]. Additionally, ZEMA is sufficiently funded to carry out its work by the government of Zambian through the Environmental Management Act No. 12 of 2011 [58]. Zambia has also benefited from the World Bank's support for the project on environmental safeguards enforcing regulations for mineral extraction [59]. In contrast, in the DRC, despite the outcry by national members of parliament on environmental pollution from mining companies that has led to numerous revisions of the mining code to mitigate the situation up to the current “Code Minier” amended and completed by decree *no.*° 18/024 of June 8, 2018 [60], neither mining companies have hardly complied with the environmental regulatory requirements, nor the government systematically monitored the industries' implementation of the environment assessment protocols [7, 44].

The findings of this study were shared with all stakeholders including local communities, government officials, research and high learning institutions, mine and metallurgic industries, NGOs, CBOs, churches, and other interested stakeholders during awareness workshops in the two countries, and the outcome recommendations cleared indicate that the study findings provide a foundation for urgent policy regulation reinforcement. Interventions that span the entire central African Copperbelt region on sustainable governance of mining and metallurgic industry exploitation are required. In this study, we emphasize the need for rigorous administration and monitoring of environmental protection laws to ensure sustainable management of environmental pollution in addition to making awareness campaigns in the region. There are also several other management strategies that will need to be explored. It is well established that the uptake and distribution of heavy metals considerably vary across and within crop species [61, 62], and this depends on their morpho-physiological differences such as cuticle layer texture and composition, activity of antioxidative enzymes, uptake speciation, and plant physiological maturity [63]. Whether this is true in our study needs further investigations. Crop breeding programs should urgently be initiated in both the two countries to select cultivars with the lower accumulation of heavy metals for wider cultivation in the affected ecosystems. In addition, implementation of other management strategies have showed better results such as land remediation methods that might include physical land isolation and containment of acid-producing materials, farmers' land-use restriction to growing crops, and *in situ* soil toxicity reduction by flooding, for example, neutralizing materials as pyrite to cut off or reduce impacts of toxic elements, stopping the development of acidic conditions, and preventing mobilization of metals [64, 65].

## 6. Conclusion

Our results have conclusively provided compelling evidence of mining and metallurgic operations discharging large amounts of toxic metals in the ecosystem causing severe environmental pollution of water, food crops, and ambient air in the central African Copperbelt region that is negatively impacting the well-being of the surrounding communities. We observed that children were more vulnerable than adults to pollutants. Besides exploiting and processing minerals solely for financial gain and the governments prioritising only on tax contribution by miners, they have tremendously failed to pay more attention to regular mechanisms and extent of monitoring and enforcing the safety measures and laws for environmental protection. There are therefore urgent needs for stringent legislative and policy reinforcement of industrial activities for sustainable exploitations to alleviate negative impacts on environmental protection, food and nutrition safety, and population health. However, there exist also other potentially effective techniques for heavy metal management—such as cultivation of heavy metals tolerant crop species and land remediation methods—that are readily available for use. These should be adopted and integrated into the overall management strategies for heavy metals in the region to protect the local communities and ensure their sustainable livelihood.

## Figures and Tables

**Table 1:** Population perception of the impacts of mining and metallurgic industries on the livelihood and health of different locations, artisanal miners, traders, price negotiators, and NGOs in Zambia and the DR Congo.

Salient mining problems discussed with urban and rural residents	ZAM (*n* = 180)		DRC (*n* = 318)		Community reaction to mining and metallurgic industry pollution	Local health services		NGOs		dMiners/traders/mineworker union	
	% yes	% no	% yes	% no		ZAM	DRC	ZAM	DRC	ZAM	DRC
Loss of agricultural land	68.0	32.0	100	0.0	Pollution has deteriorated our land			●c	○		
Decrease in wild food such as mushrooms, and caterpillars	100	0.0	100	0.0	We now travel several kms to get some food from the wild			●	○		
Water pollution from mine discharge	56.0	44.0	100	0.0	The water used is of poor quality	●	○	●	○	●	○
Soil degradation from mine pollution	85.0	15.0	100	0.0	Crop yield has decreased	●	○	●	○	●	○
Drying of some streams	24.0	76.0	35.0	65.0	Several streams have disappeared	●	○	●	○		
Health issues among residents	100	0.0	100	0.0	We suffer from numerous illnesses	●	○	●	○	●	○
Alternative to water at homestead	0.0	100	0.0	100	There is nowhere water is pure					●	○
Normal life if water source was cut off or contaminated	0.0	100	0.0	100	This is the only water we have					●	○
Food consumption grown around homestead/village?	100	0.0	100	0.0	Because of poverty, everyone has a garden to sustain livelihood					●	○
Role of mining in livelihood	100	0.0	100	0.0	We except a lot from mine industries					●	○

^a^Percentage rounded to the nearest one of residents acknowledging the issue (yes) and used to deduct the no respondents. ^b^Cancer, diarrhea, respiratory issues, eye and throat irritation, skin tingling, fatigue, and congenital defects. ^c^Recognition of the problem being present in Zambia (ZAM) (●) and in the Democratic Republic of Congo (DRC) (○), except otherwise stated. ^d^Artisanal miners and traders do not perceive any illness that could arise from their activity.

**Table 2:** Most salient negative impacts of the mines and metallurgic industries among residents in surveyed locations in Zambia and the DR Congo.

Noise pollution Modern mines use heavy and loud equipment that frequently causes blasting that imposes intolerable stress upon the local people	Indigenous rights Local people do not have recognized land rights and mines frequently hide behind this to apply double standards in treating them	Displacement Displacement of people including forced resettlement in areas without adequate resources remains a common feature of mining development	Human rights violations Opposition to mine operations is suppressed using force and military or police deployment, violating human rights through torture, arrest, and harassment

Employment and livelihoods One of the positive incentives to allow mining operations is employment that often fails to materialize causing high poverty among the local communities	Treatment of women and children Often, mining jobs are restricted to men, thus alienating children's and women's livelihoods such that unaccompanied men suffer severe prostitution and high incidence of HIV and AIDS	Health hazards Contamination of available resources, as well as soil and air, contributes to increased levels of toxic build-up in peoples' bodies. Several chronic respiratory issues are widespread, particularly in children and old people	Environmental degradation Mining exploitation of natural resources has taken a toll on the quality of land, water availability and quality, forest and soil resources, and rainfall patterns, undermining local livelihoods in the process

^a^Nearly all respondents recognized that industrial operations have impacted negatively on their household livelihoods with pollution of water, degradation of farmland, reduction in vegetation growth and forest natural resources, and population's danger with health issues.

## Data Availability

The data used to support this study are available in the Department of Plant and Environmental Science, School of Natural Resources, Copperbelt University, P. O Box 21692, Jambo Drive, Riverside, Kitwe, Zambia, and in the Unit of Toxicology and Environment, School of Public Health, Faculty of Medicine, University of Lubumbashi, Democratic Republic of Congo.

## Abbreviations

CBO: Community-based organizations
DRC: Democratic Republic of Congo
FANR: The Food, Agriculture and Natural Resources of SADC
FAO: Food and Agriculture Organization of the United Nations
FDI: Foreign direct investment
Gécamines: Générales des carrières et des mines
GNP: Gross national product
KCM: Konkola copper mines
NGO: Non-governmental organizations
SADC: Southern African Development Community
UNEP: United Nations Environment Program
WHO: World Health Organization
ZEMA: Zambia Environmental Management Agency.

## Supplementary Materials

Supplementary Materials Table 1. Concentration of trace elements (*μ*g/L) in water samples from Zambia and the DR Congo. All values are *μ*g/L, except where indicated. LOD is the limit of detection, LOQ is the limit of quantification, WHO is the upper limit for drinking water according to World Health Organization guidelines (not available for Co); P5–P95 are 5–95th percentiles; min is the minimum value; MAX is the maximum value; ratio is P50 of DRC divided by P50 of Zambia; ^*∗*^*p* value for the difference between Zambia and DRC (Mann–Whitney test); values in italics are imputed values (LOD/2). Table 2. Concentration of trace elements (*μ*g/g) in food crops from Zambia and the DR Congo. All values are *μ*g/g dry weight except where indicated. LOD is the limit of detection. LOQ is the limit of quantification. WHO is the upper limit for food crops according to World Health Organization guidelines (not available for Co); P5–P95 are 5–95th percentiles; min is the minimum value. MAX is the maximum value; median ratio is P50 of DRC divided by P50 of Zambia; ^*∗*^*p* value for the difference between Zambia and DRC (Mann–Whitney test); values in italics are imputed values (LOQ/2). Supplementary Table B. Spearman's correlations of concentrations of 9 trace metals in vegetable samples^a^ from Zambia and the DR Congo. ^a^Concentrations of nine trace elements were generally weak or nonexistent for samples obtained from Zambia and high for those obtained from the DR Congo. Supplemental Table D. Geometric means (GMs) and their 95% confidence intervals of trace metal concentrations (mg/kg dry weight) in food crops in Zambia (Z) and the DR Congo (C) and according to distance from mining (far or near). GM ratios between relevant columns are indicated and, if significant (*p* < 0.05), the cells are filled green (Mann–Whitney test) or yellow (Dunn's post hoc test).
