# Supplementary material for: Impacts of Trace Metals Pollution of Water, Food Crops, and Ambient Air on Population Health in Zambia and the DR Congo
Source: J Environ Public Health. 2022 Jul 5;2022:4515115. doi: 10.1155/2022/4515115 (PMC9277192; doi:10.1155/2022/4515115)
Supplement: Supplementary Materials — Table 1. Concentration of trace elements (μg/L) in water samples from Zambia and the DR Congo. All values are μg/L, except where indicated. LOD is the limit of detection, LOQ is the limit of quantification, WHO is the upper limit for drinking water according to World Health Organization guidelines (not available for Co); P5–P95 are 5–95th percentiles; min is the minimum value; MAX is the maximum value; ratio is P50 of DRC divided by P50 of Zambia; ∗p value for the difference between Zambia and DRC (Mann–Whitney test); values in italics are imputed values (LOD/2). Table 2. Concentration of trace elements (μg/g) in food crops from Zambia and the DR Congo. All values are μg/g dry weight except where indicated. LOD is the limit of detection. LOQ is the limit of quantification. WHO is the upper limit for food crops according to World Health Organization guidelines (not available for Co); P5–P95 are 5–95th percentiles; min is the minimum value. MAX is the maximum value; median ratio is P50 of DRC divided by P50 of Zambia; ∗p value for the difference between Zambia and DRC (Mann–Whitney test); values in italics are imputed values (LOQ/2). Supplementary Table B. Spearman's correlations of concentrations of 9 trace metals in vegetable samplesa from Zambia and the DR Congo. aConcentrations of nine trace elements were generally weak or nonexistent for samples obtained from Zambia and high for those obtained from the DR Congo. Supplemental Table D. Geometric means (GMs) and their 95% confidence intervals of trace metal concentrations (mg/kg dry weight) in food crops in Zambia (Z) and the DR Congo (C) and according to distance from mining (far or near). GM ratios between relevant columns are indicated and, if significant (p < 0.05), the cells are filled green (Mann–Whitney test) or yellow (Dunn's post hoc test). [file 4515115.f1.zip › 4515115.f1/Supplementary data Table B.docx]

**Supplementary Table B.** Spearman correlations of concentrations of 9 trace metals in vegetable samples^a^ from Zambia and the DR Congo

| ALL (n=273) | Mn | Co | Ni | Cu | Zn | As | Cd | Pb | U |
| --- | --- | --- | --- | --- | --- | --- | --- | --- | --- |
| Mn | 1.00 | 0.06 | 0.08 | -0.02 | 0.19 | -0.06 | 0.06 | 0.00 | -0.03 |
| Co |  | 1.00 | 0.27 | 0.83 | 0.46 | 0.54 | 0.62 | 0.72 | 0.64 |
| Ni |  |  | 1.00 | 0.30 | 0.11 | 0.19 | 0.36 | 0.37 | 0.31 |
| Cu |  |  |  | 1.00 | 0.41 | 0.54 | 0.61 | 0.71 | 0.73 |
| Zn |  |  |  |  | 1.00 | 0.40 | 0.50 | 0.45 | 0.34 |
| As |  |  |  |  |  | 1.00 | 0.56 | 0.60 | 0.40 |
| Cd |  |  |  |  |  |  | 1.00 | 0.74 | 0.51 |
| Pb |  |  |  |  |  |  |  | 1.00 | 0.58 |
| U |  |  |  |  |  |  |  |  | 1.00 |
|  |  |  |  |  |  |  |  |  |  |
| ZAMBIA (n=128) | Mn | Co | Ni | Cu | Zn | As | Cd | Pb | U |
| Mn | 1.00 | 0.10 | 0.02 | -0.06 | 0.23 | 0.00 | 0.24 | 0.00 | -0.08 |
| Co |  | 1.00 | 0.00 | 0.63 | 0.21 | 0.18 | 0.30 | 0.43 | 0.45 |
| Ni |  |  | 1.00 | 0.12 | -0.09 | -0.11 | 0.20 | 0.21 | 0.22 |
| Cu |  |  |  | 1.00 | 0.13 | 0.20 | 0.51 | 0.57 | 0.61 |
| Zn |  |  |  |  | 1.00 | 0.06 | 0.27 | 0.14 | 0.09 |
| As |  |  |  |  |  | 1.00 | 0.07 | 0.15 | 0.21 |
| Cd |  |  |  |  |  |  | 1.00 | 0.43 | 0.38 |
| Pb |  |  |  |  |  |  |  | 1.00 | 0.41 |
| U |  |  |  |  |  |  |  |  | 1.00 |
|  |  |  |  |  |  |  |  |  |  |
| DRC (n=145) | Mn | Co | Ni | Cu | Zn | As | Cd | Pb | U |
| Mn | 1.00 | 0.06 | 0.17 | 0.03 | 0.14 | -0.08 | -0.07 | -0.01 | 0.04 |
| Co |  | 1.00 | 0.33 | 0.93 | 0.50 | 0.53 | 0.67 | 0.84 | 0.77 |
| Ni |  |  | 1.00 | 0.35 | 0.18 | 0.16 | 0.29 | 0.28 | 0.29 |
| Cu |  |  |  | 1.00 | 0.49 | 0.62 | 0.67 | 0.83 | 0.81 |
| Zn |  |  |  |  | 1.00 | 0.47 | 0.60 | 0.61 | 0.47 |
| As |  |  |  |  |  | 1.00 | 0.59 | 0.66 | 0.48 |
| Cd |  |  |  |  |  |  | 1.00 | 0.74 | 0.61 |
| Pb |  |  |  |  |  |  |  | 1.00 | 0.75 |
| U |  |  |  |  |  |  |  |  | 1.00 |

^a^concentrations of nine trace elements were generally weak or non-existent for samples obtained from Zambia, and high for those obtained from the DR Congo.
